# Engineered Janus probes modulate nucleic acid amplification to expand the dynamic range for direct detection of viral genomes in one microliter crude serum samples†
†Electronic supplementary information (ESI) available. See DOI: 10.1039/c7sc03994h


**DOI:** 10.1039/c7sc03994h

**Published:** 2017-10-27

**Authors:** Yue Zhao, Feng Chen, Jing Qin, Jing Wei, Wenhua Wu, Yongxi Zhao

**Affiliations:** a Key Laboratory of Biomedical Information Engineering of Education Ministry , School of Life Science and Technology , Xi’an Jiaotong University , Xianning West Road , Xi’an , Shaanxi 710049 , P. R. China . Email: yxzhao@mail.xjtu.edu.cn; b Department of Infectious Disease , The Second Affiliated Hospital of Medical College , Xi’an Jiaotong University , Xiwu Road , Xi’an , Shaanxi 710049 , P. R. China

## Abstract

The viral genome load in diverse clinical samples varies over several orders of magnitude (*e.g.* 1–10^4^ copies per μL), thus a dynamic range-extended and sensitive analysis method is highly desired. However, existing well-developed nucleic acid amplification systems always suffer from either a limited dynamic range or modest sensitivity for analysis of these samples. Herein, we propose a general engineered Janus probe to modulate the thermodynamics and kinetic properties of the amplification reaction. Through rational regulation, the Janus system improves the performance by both reducing the background and enhancing the signal, expanding the operative dynamic range by 2 orders of magnitude. This proposed approach achieves a detection limit for hepatitis B virus (HBV) DNA of down to 3 copies and can be successfully applied for direct quantification of the HBV genome in one microliter crude serum samples without any pretreatment. The results are consistent with clinical diagnosis and hold considerable potential to discriminate healthy volunteers and patients at different disease stages. Whereas, following the same operation, the representative well-developed system provided serious false-negative results using such trace amounts of samples from clinically confirmed positive patients.

## Abstract 2

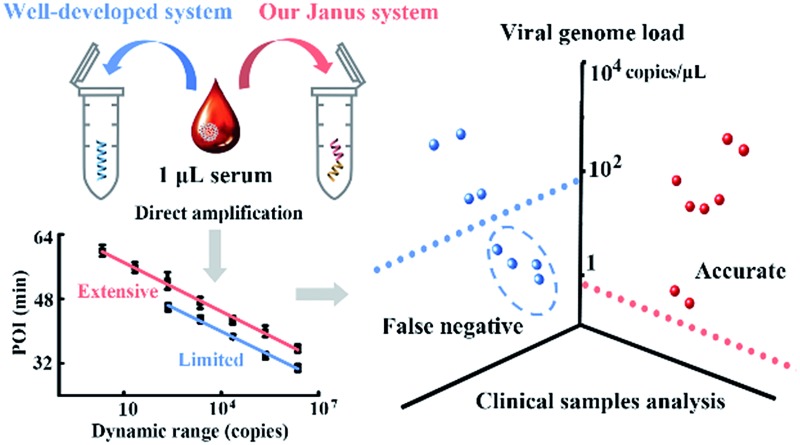
Janus probes were designed to expand the dynamic range of amplification for viral genome quantification in 1 μL crude serum.

## Introduction

Viral genome load is an objective measurement for diagnosis, assessing severity and monitoring progression of viral infectious diseases.^1^ Due to the diversity and complexity of clinical samples, the amount of viral genome spans several orders of magnitude (*e.g.* 1–10^4^ copies per μL),^2^ thus requiring analysis methods with an extensive dynamic range and predominant sensitivity. Nucleic acid amplification efficiently accumulates millions of target sequences from the trace template and has emerged as a promising new tool for clinical analysis.^3^–^5^ However, the undesirable nonspecific amplification frequently encountered hinders detection of a low-concentration target, resulting in a limited dynamic range and inadequate sensitivity.^6^,^7^ Typically, the polymerase chain reaction (PCR), which has become a routine tool to amplify target DNA fragments, commonly has chimeric artifacts such as primer-dimers.^8^–^10^ To mitigate these limitations, primers with optimized amplified loci, an appropriate annealing temperature, precise structure^11^,^12^ or reduced concentration^13^,^14^ are frequently involved. As another alternative, isothermal nucleic acid amplification has attracted a great deal of attention for its ability to generate large quantities of DNA amplicons at a constant temperature.^15^–^17^ This simple feature allows it to outperform PCR in operation but increases the risk of nonspecific amplification to be even more severe than that for PCR. Without cyclic denaturation–renaturation procedures, spurious amplicons are accumulated during continuous reactions, resulting in a worse dynamic range and a corresponding loss in sensitivity.^18^,^19^ Substantial effort thus far has been devoted to regulating the dynamic range *via* enlarging the difference between the signal and background (denoted by the signal-to background ratio, SBR), and target independent DNA synthesis was demonstrated to be ubiquitous among amplifications that are synergistically catalyzed by restriction endonucleases and polymerase (RE-pol).^20^,^21^

For further exploration of nucleic acid amplification, the well-developed loop-mediated isothermal amplification (LAMP) is chosen as it only requires one polymerase. Though circumventing RE-pol-based interferences,^22^ LAMP is still unable to exclude undesired background amplifications entirely.^23^,^24^ Utilizing six primers to recognize eight distinct regions on the target DNA, LAMP is obliged to obey primer design principles about primer length and hybridization site in order to guarantee a feasible amplification. These stringent rules limit the variability of the primers and pose a challenge for adjusting amplification based on primer optimization.^25^,^26^ Moreover, coupled with numerous sets of high-concentration primers, LAMP is much more vulnerable to nonspecific amplification induced by primer-dimers. In response, a variety of approaches have been explored to expand the dynamic range and distinguish true from false amplicons in LAMP, such as invoking a strand-displacement probe,^24^,^27^ touchdown temperature and additives such as dimethyl sulfoxide.^28^ Due to nonspecific amplification being contingent upon multiple parameters, the emerging need for a general approach to modulate LAMP remains to be addressed. To improve the dynamic range and sensitivity, our implementation obviously consists of a reduction in the background and an increase in the signal. Inspired by the prominent performance of common sequence (CS)-tagged primers for alleviating biased genome coverage,^29^ we suppose that this concept may also work for the inhibition of primer-dimers in isothermal amplifications. Meanwhile, similarly to inner primers hybridizing with the stem-loop region of cauliflower-like DNA products, loop primers also hold the potential to initiate self-primed extensions, causing multiple positive feedback loops and further accelerating amplifications *via* simply linked distinct sequences.

Motivated by the above consideration, we proposed a general approach to engineer Janus probes with a specially designed terminal for modulating nucleic acid amplification. We designed this kind of bifunctional probe by gaining fundamental insight into the thermodynamics and kinetic properties of the reaction and this reveals that the dynamic range and efficiency of nucleic acid amplification could be reasonably regulated. With the normalized terminal, the Janus probe has features on both sides so that it can both participate in the reaction to inhibit primer-dimers and contribute to promoting amplification synchronously. Due to the controllability and improvement of the amplification reaction, as little as 3 copies of hepatitis B virus (HBV) DNA can be detected *via* the Janus probe system. Furthermore, we applied this proposed approach in one microliter crude serum samples without any pretreatment. The HBV genome load was directly and accurately determined and the results are consistent with clinical diagnosis. According to the clinical index of 1 copy per μL, which is reported to be the baseline of HBV genome load present in the blood of patients with HBV infection, the Janus system exhibited a clear discrimination between healthy volunteers and patients at different disease stages, whereas the representative existing well-developed system presented serious false-negative results for clinically confirmed positive patients with such trace amounts of samples.

## Results and discussion

**Fig. 1:**
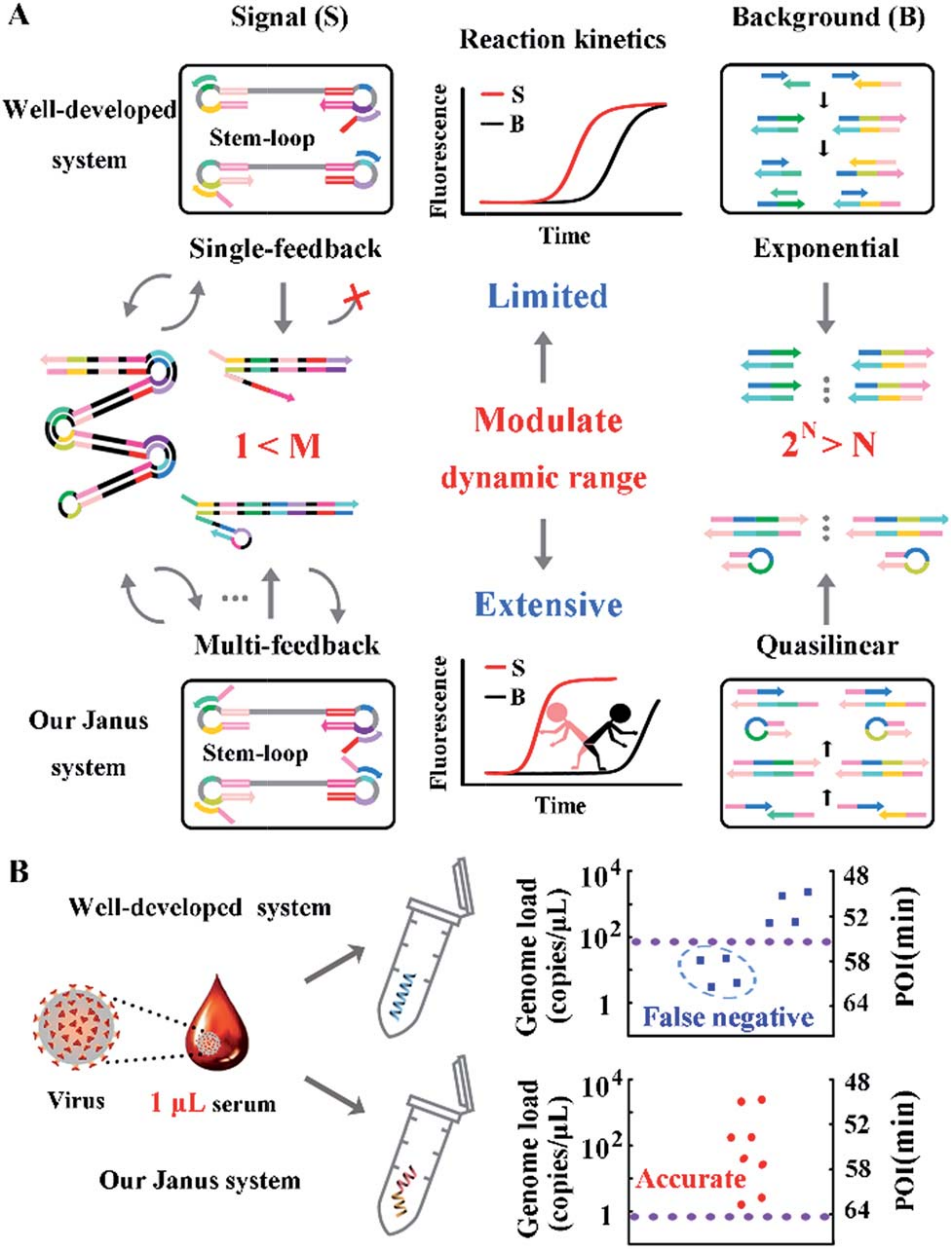
(A) Schematic illustration of engineered Janus probes to modulate nucleic acid amplification. (B) Clinical analysis of HBV in one microliter crude serum samples *via* direct amplification.

The probes involved in the nucleic acid amplification system consist of a pair of inner primers and a pair of loop primers as illustrated in Fig. 1A. For ease of presentation, eight distinct regions are labeled with different colors. In the existing well-developed system, the loop primers disrupt or destabilize double stranded DNA to assist the inner primer’s binding or self-priming and initiate new parallel amplification pathways, thus indeed promoting the accumulation of cauliflower-like structures. However, due to the lack of a loop-back feature, the loop primers are not competent at self-primed DNA synthesis, thus their 5′ terminal plays no role in the auto-cycling amplification to regenerate stem-loop DNA. Meanwhile, because the primers are present at high concentrations, even weak interactions can occur and give rise to background amplification. Following the binding of a new primer to any transiently single-stranded region, the non-specific products will be exponentially accumulated, resulting in a poor dynamic range and a loss of sensitivity for analysis. Based on this well-developed system, our system is introduced with a pair of bifunctional Janus probes instead of ordinary loop primers. To examine the effect of the Janus probes on the reaction kinetics, we systematically investigated the Gibbs free energy of DNA with a stem-loop structure and proposed a general approach to this kind of ingeniously designed probe. Specifically designed sequences are tagged to the 5′ terminal of original loop primers, providing a controllable and tunable amplification system. Normalized terminal loop primers offer a new ability to self-initiate strand displacement DNA synthesis, generating multiple positive feedback loops to the upstream reaction and enhancing the amplification efficiency. In addition, due to the complementary sequences incorporated into the potential primer-dimers derived from inter-primer extension, entropy-driven intramolecular interactions are prone to cause nonspecific amplification products to form hairpin structures. Thus occasional released single strand artifacts by primer extension may outcompete the annealing of primers, prevent further nonspecific amplification and suppress the background down to a quasilinear mode. Using this two-pronged strategy *via* Janus probes we regulated the signal and background simultaneously in the nucleic acid amplification system and expanded the dynamic range by 2 orders of magnitude. We further demonstrated the feasibility of this approach for direct HBV quantification in one microliter crude serum samples. The results of all the samples using our Janus system are in accordance with clinical diagnostics, whereas the existing well-developed LAMP system presents serious false negative results (Fig. 1B).

**Fig. 2:**
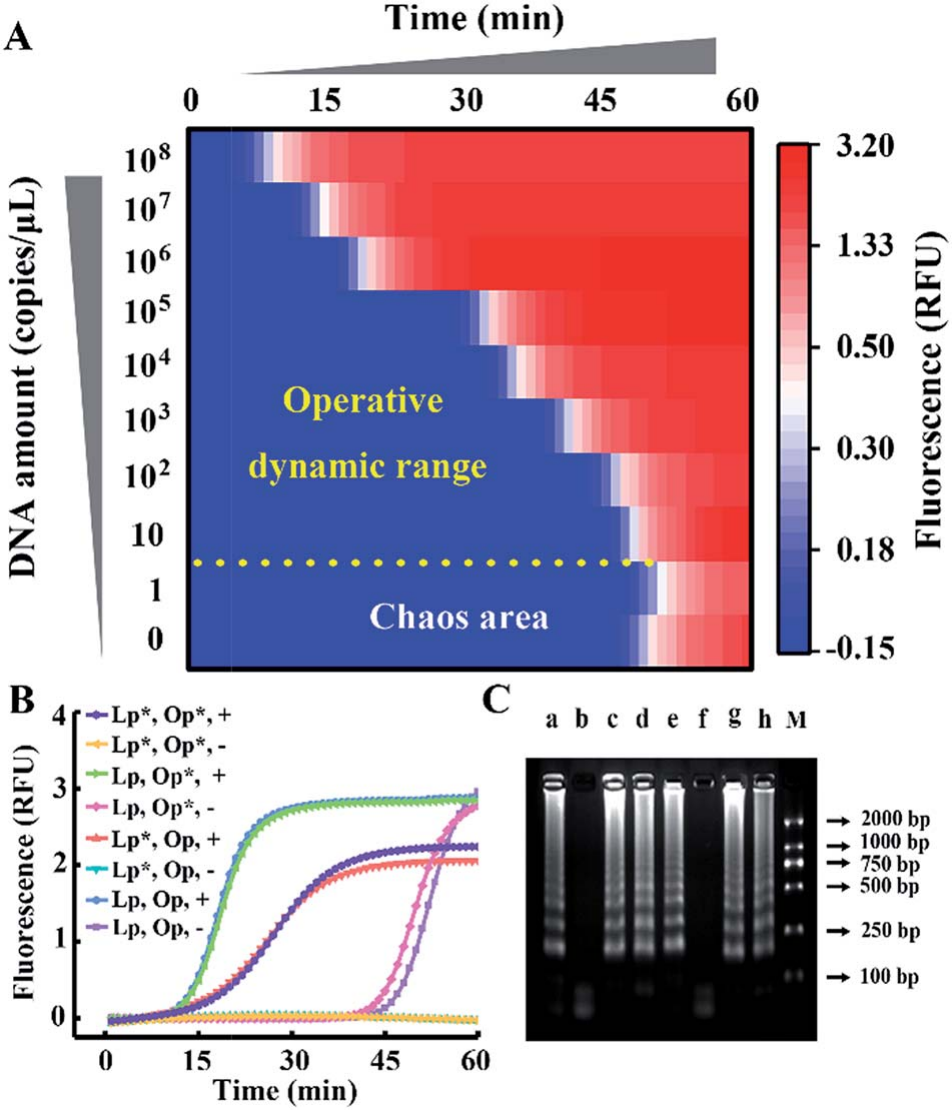
(A) The dynamic range and sensitivity of the quantification of standard template DNA with the well-developed LAMP system. (B) Real-time fluorescence signals of the LAMP reactions with different probe sets. The CS tagged probes are marked by an asterisk (*) to distinguish them from standard probes. Plus (+) and minus (–) signs represent amplifications in the presence of 10^7^ copies per μL of template DNA and the corresponding background controls without template DNA, respectively. (C) Gel electrophoresis analysis: lanes a to h correspond to the reactions in (B) from the top down. Lane M shows the DNA ladder markers ranging from 100 to 2000 bp.

We take HBV, which accounts for the majority of inflammatory liver disease and infects nearly 350 million people worldwide,^28^ as a model analyte for exploration of our proposed approach. Through alignment of HBV genomic sequences from the National Center for Biotechnology Information nucleotide sequence database (GenBank), the partial HBV gene (Fig. S1†) was directly cloned into a plasmid as standard template DNA. Standard LAMP primers were designed by the PrimerExplorer V5 program at the website ; http://primerexplorer.jp/e/, and applied in the well-developed system to quantify the amount of template DNA. However, due to the serious nonspecific amplification, the signal of the low-concentration target shows large fluctuations and a chaos area in which the signal cannot be distinguished from the background. For a direct display of the results, the point of inflection (POI), defined as the time corresponding to the maximum slope in the real-time fluorescence curve (Fig. S2†), is presented as the site where the color drastically changes from blue to red (Fig. 2A). In this heat map, the area above the dashed line is the operative dynamic range, corresponding to an amount of template DNA from 10 to 10^8^ copies per μL. The data in the chaos area always lack reproducibility and make no sense for quantification, thus limiting detection for targets with a low concentration. Inspired by the prominent performance of CS tailed primers for alleviating biased genome coverage, we suppose this concept may also work for the inhibition of primer-dimers in isothermal systems. Apart from the inner primers, whose 5′ terminal sequences are specific to the target, we tagged all other primers in probe sets with a 5′ common tail (marked by asterisks in Fig. 2B). Since outer primers (abbreviated as Op in Fig. 2B) are not involved in the cyclic amplification stage of the LAMP reaction, they have little adverse effect on reaction regulation, as shown in Fig. 2B. As the major cause of nonspecific amplification, loop primers (abbreviated as Lp in Fig. 2B) are tagged with CSs at the 5′ terminal and inhibit the background as significantly as the all primer-tagged set (outer and loop primers). Furthermore, the amplification performances were investigated by agarose gel electrophoresis (Fig. 2C). The obvious spurious amplicon bands observed in the untagged set and outer primer-tagged set are almost entirely eliminated in the loop primer-tagged set.

**Fig. 3:**
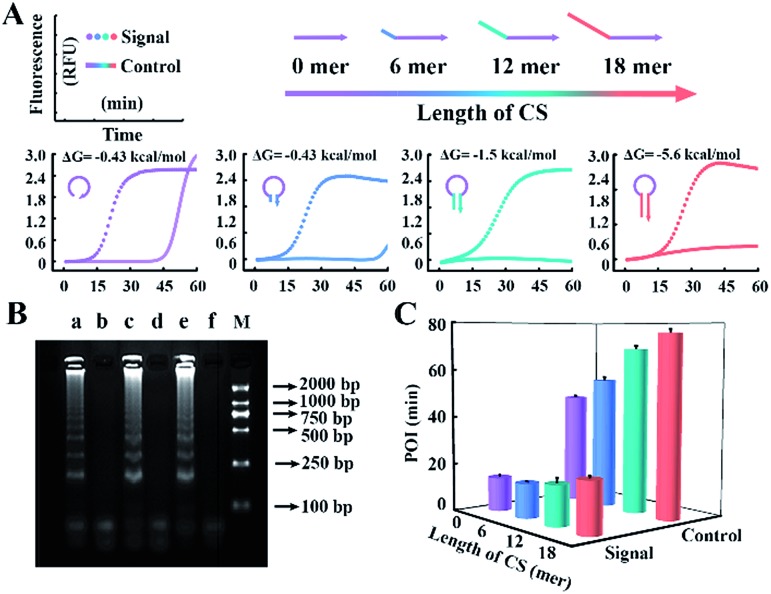
(A) Real-time fluorescence analysis of the reaction involving CS-tagged probes with varying length from 0 to 18 mer (highlighted in different colors). The dotted line and straight line represent the signal from 10^7^ copies per μL of template DNA and the corresponding background control without template, respectively. The predicted Gibbs free energy and DNA structure of potential primer-dimers are also revealed. (B) Gel electrophoresis analysis for the signal of the 6 mer (lane a), 12 mer (lane c) and 18 mer (lane e) CS tagged-probe sets and their corresponding background controls (lane b, lane d and lane f) in (A). Lane M shows the DNA ladder markers ranging from 100 to 2000 bp. (C) Signal and background of the amplification reactions regulated by various CS-tagged primers.

According to the results above, CSs with various lengths are tagged at the 5′ terminal of loop primers to investigate the performance for amplification. The Gibbs free energies of the potential primer-dimers are predicted by UNAfold software online (http://sg.idtdna.com/UNAFold) and the duplex stability of hairpin DNA is investigated by melting curve analysis (Fig. S3†). CSs with lengths from 6 mer to 18 mer are tagged at the end of loop primers and all reactions suppress the background, which is confirmed by real-time fluorescence (Fig. 3A) and electrophoresis analysis (Fig. 3B). Increasing the length of the CS increases the stability of the entropy-driven intracellular hairpin of potential primer-dimers, which reduces the background. However, long-chain probes accompanied by a complex secondary structure often cause steric hindrance and then disturb the signal. Thus the POI is adopted to evaluate the signal and background of amplification among various CSs. As can be seen in Fig. 3C, the CS with a 12 mer tail is selected as it has the highest SBR.

**Fig. 4:**
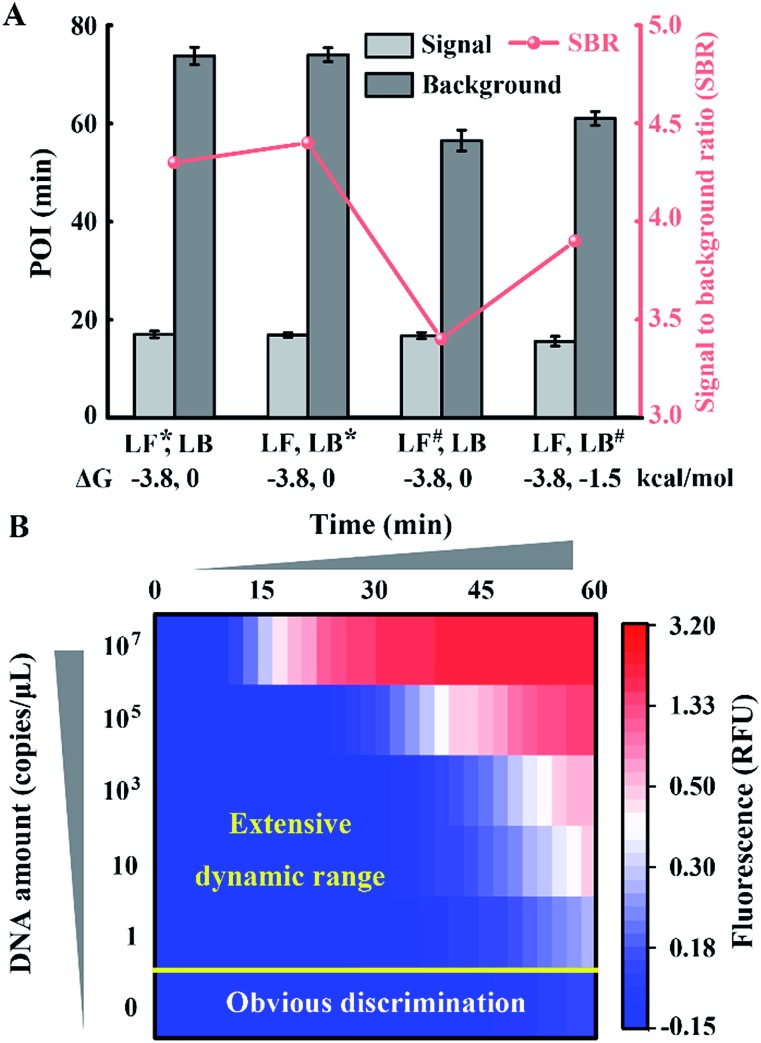
Function of the normalized probes on the regulation of the amplification system. (A) Structure prediction and SBR evaluation of various probe sets including (LF*, LB), (LF, LB*), (LF^#^, LB) and (LF, LB^#^). Asterisks (*) indicate the normalized primers and the pound signs (#) indicate the random sequence tagged primers. (B) The dynamic range and sensitivity of the quantification for standard template DNA with the normalized probe set.

To obtain appropriate regulation, the sequence of common tails is always required to be optimized carefully. Besides avoidance of complex self-secondary structures, interactions with other probes are also forbidden. Therefore, we try to propose a general approach to CSs. To guarantee maximum system stability, we pick CSs from sequences of the well-developed system. Maintaining the original structure of one loop primer, we simply add its identical terminal sequence at the 5′ terminal of the other loop primer. So complementary fragments can also be introduced into the possible primer-dimers by the above normalization. As shown in Fig. 4A, various combinations of tagged or untagged forward loop primers and backward loop primers are selected for further investigation. We designed two probe sets with loop primers normalized to either a forward loop primer (denoted as LB*) or a backward loop primer (denoted as LF*). The POI of both the signal and background reaction is presented to evaluate the SBR of all the probe sets. Notably, normalized primers play a vital role in the elimination of background amplification, as shown in Fig. S4 and S5.† To exclude the possibility that the inhibition of the background is only due to the steric hindrance of the extra tagged tails, we confirm the structures of the normalized probes *via* prediction of Gibbs free energy. For the sequences of the well-developed system generated *via* proprietary software, the truncated sequences are also compatible with the original system. Thus the normalized probes in both sets possess similar structures to the standard loop primer in the well-developed system. To further demonstrate this conclusion, random sequences with similar structures (denoted as LF^#^) or even more complex secondary structures (denoted as LB^#^) are appended to the standard loop primer to evaluate steric hindrance. In accordance with expectations, only the normalized probes exhibit a predominant inhibition of the background (Fig. 4A). The quantification results are shown in Fig. 4B, in which the background has been restrained to a large extent. Thus we obtained a distinct discrimination between the blank background and the low-concentration target and expanded the dynamic range down to 1 copies per μL (equivalent to 30 copies per reaction).

**Fig. 5:**
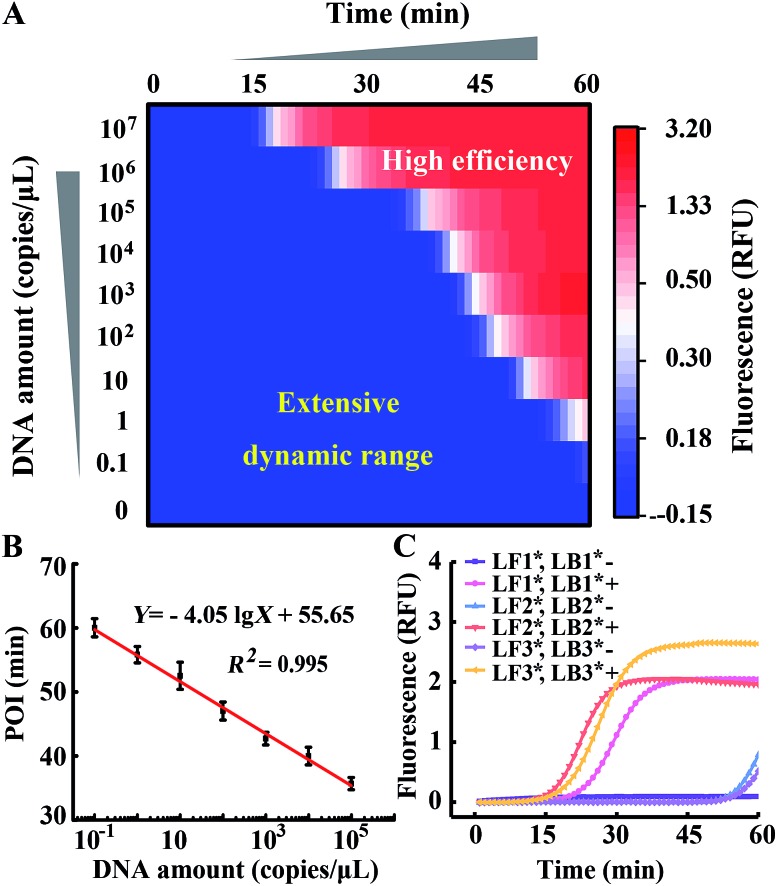
(A) The dynamic range and sensitivity of the quantification of template DNA in the Janus system (LF1*, LB1*). (B) The linear correlation of the POI with DNA amount in the range of 0.1–10^5^ copies per μL. (C) Real-time fluorescence signals of LAMP reactions with different Janus probe sets (denoted by asterisks).

Comparing Fig. 4 with Fig. 1, we indeed inhibit background amplification by normalization of loop primers, but the target amplification kinetics also fell into a decline, which is shown by the dominant hue of the signal changing from red to white. Having a similar function to the inner primer, loop primers also anneal to the loop region of stem-loop structures and are able to initiate self-primed amplifications (Fig. S6†). We tried to invoke a loop primer *via* imitating an inner primer’s structure, activating self-initiated extension to improve auto-cycling amplification efficiency. Tagged by sequences the same as the loop-back region of inner primers, the normalized loop primer is confirmed to be effective at increasing the reaction speed for amplification (Fig. S7†). Taking both sides into consideration, the Janus system has been developed to both inhibit the background and improve the signal (Fig. S8†). In this proposed approach, one loop primer of the probe set is normalized to the inner primer and creates multiple positive feedback loops to accelerate amplification. Then the other loop primer of the probe set is tagged by a CS of the normalized loop primer to repress the background. As shown in Fig. 5A, amplification in the presence of template DNA of a specific amount can be distinctly distinguished from amplification in the presence of different specific amounts of template DNA in this heat map. Through modulating nucleic acid amplification *via* engineered Janus probes, we obtained an excellent dynamic range without compromising on the reaction kinetics. The quantitative analysis of standard template DNA was subsequently evaluated. Fig. 5B plots the relationship between the POI and the amount of template DNA in the range of 0.1–10^8^ copies per μL. It can be seen that there is a linear correlation between the POI and the logarithm of DNA amount (0.1 to 10^5^ copies per μL), and that the detection limit is equivalent to 3 copies per reaction. Systematic generalization was further proved by tagging different sequences of forward inner primer or backward inner primer to normalize the loop primers. Janus set 1 (LF1*, LB1*) is normalized to the 5′ middle sequences of forward inner primers, Janus set 2 (LF2*, LB2*) is normalized to the middle sequences of backward inner primers and Janus set 3 (LF3*, LB3*) is normalized to the 5′ terminal sequences of forward inner primers. As shown in Fig. 5C, all Janus systems exhibit a consistent and satisfactory suppression of the background with rapid reaction kinetics of signal amplification.

**Fig. 6:**
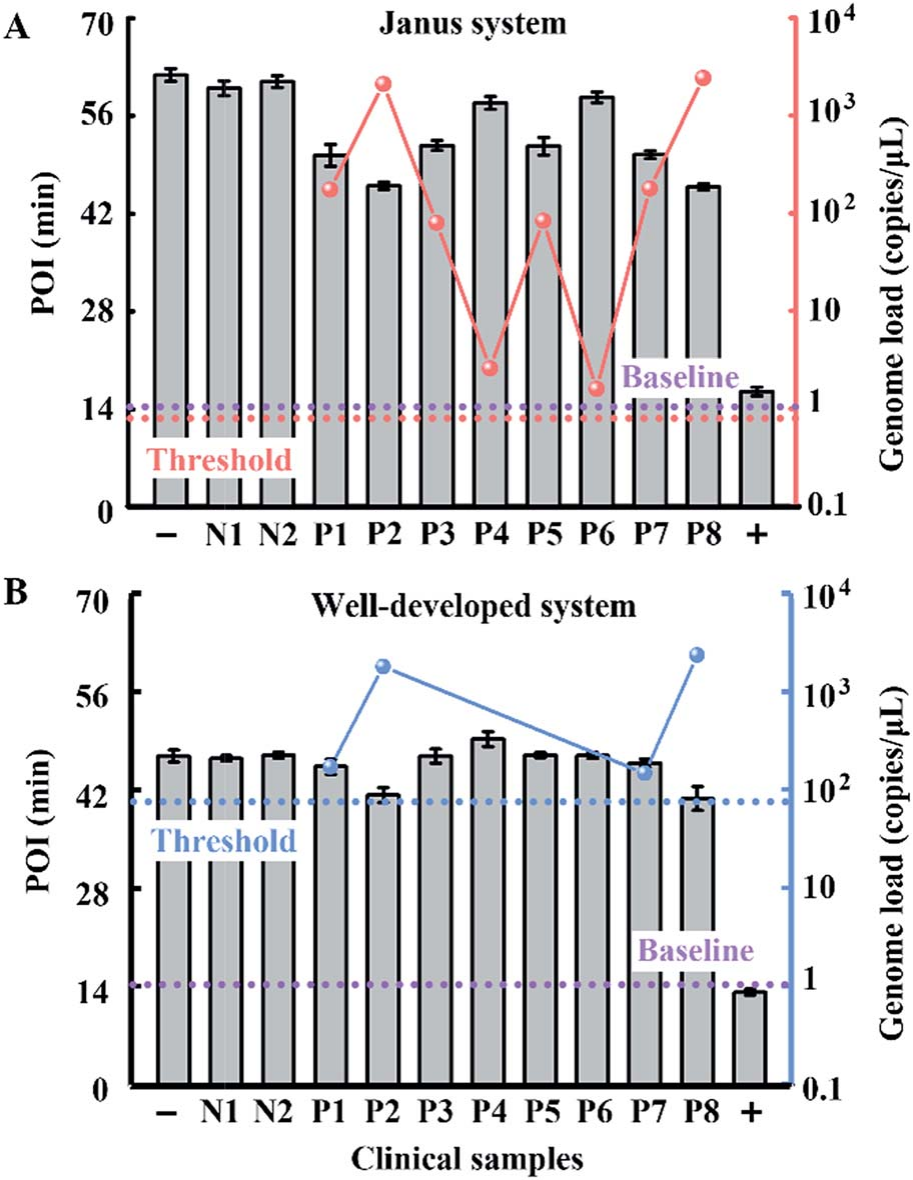
Clinical assay performance of HBV genome load quantification in crude serums of patients and healthy volunteers *via* our Janus system (A) and the well-developed system (B). The red dots represent the genome load quantified by the Janus system and the blue dots represent the genome load detected by the well-developed system. Plus and minus signs represent the positive control in the presence of 10^7^ copies per μL of standard template DNA and the corresponding negative control without the template. N1 and N2 represent normal samples from healthy volunteers and P1–8 represent samples from clinically confirmed positive patients. According to the dynamic range and the amount of added serum of the two systems, the threshold values are calculated to be 0.9 (dashed line in red) and 90 copies per μL (dashed line in blue), respectively. The baseline (dashed line in purple) is defined as 1 copy per μL.

To evaluate the performance of our strategy in clinical samples, the HBV genome load in 1 microlitre of the serum (matrix interference is proven to be negligible as shown in Fig. S9†) of 2 healthy volunteers and 8 patients (Patient 2 and Patient 8 are diagnosed with carcinoma and the other 6 patients are diagnosed with cirrhosis) was directly tested without any sample pretreatment. Due to the extensive dynamic range of our Janus system, all the serum samples from patients even with a low load of HBV genome are quantified to be above baseline (1 copy per microliter of blood, which is reported to be the baseline of HBV genomic DNA present in the blood of patients with HBV infection^30^), and can be clearly distinguished from normal samples (Fig. 6A). Compared with the well-developed LAMP system, which missed 4 positive samples and presents serious false negative results (Fig. 6B), our Janus system holds considerable potential to provide diagnostic and disease progression information. We then investigated the proposed Janus system for the quantification of the HBV genome extracted by a commercial kit (Fig. S10†) and compared it with the well-developed system, the results of which agree with those of direct detection and further validate the accuracy of this proposed strategy.

## Conclusions

In this work, nucleic acid amplification was rationally regulated to obtain an extensive dynamic range covering 7 orders of magnitude (0.1–10^5^ copies per μL) as well as an improved detection limit down to 3 copies. Through fundamental insight into the Gibbs free energy of the DNA stem-loop, various functions can be integrated into the Janus system to modulate the reaction thermodynamics and kinetic properties. This twin-track design satisfies both sides to inhibit the background and promote the signal synchronously. As a test bed for evaluating our strategy, the HBV genome load is directly identified in the serum of healthy volunteers and patients. Surpassing the serious false negative results shown by the well-developed system, our Janus system shows a good consistency with clinical diagnostics. Therefore, we proposed a general approach to modulate the dynamic range of nucleic acid amplification, promising an available avenue to motivate nucleic acid amplification to be applied in clinical diagnostics with trace crude samples.

## Conflicts of interest

The authors declare no competing financial interest.

## Supplementary Material

Supplementary information
