# Supplementary material for: Engineered Janus probes modulate nucleic acid amplification to expand the dynamic range for direct detection of viral genomes in one microliter crude serum samples
Source: Chem Sci. 2017 Oct 27;9(2):392–7. doi: 10.1039/c7sc03994h (PMC5868314; doi:10.1039/c7sc03994h)
Supplement: Supplementary file 1 [file SC-009-C7SC03994H-s001.pdf]

Electronic Supplementary Material (ESI) for Chemical Science.
This journal is © The Royal Society of Chemistry 2017

Electronic Supplementary Information

Engineered Janus probes modulate nucleic acid amplification to expand the dynamic range for direct detection of viral genomes in one microliter crude serum samples

Yue Zhao,[a] Feng Chen,[a] Jing Qin,[a] Jing Wei,[a] Wenhua Wu[b] and Yongxi Zhao*

[a] Key Laboratory of Biomedical Information Engineering of Education Ministry, School of Life Science and Technology, Xi'an Jiaotong University, Xianning West Road, Xi'an, Shaanxi 710049, P. R. China.

[b] Department of Infectious Disease, The Second Affiliated Hospital of Medical College, Xi'an Jiaotong University, Xiwu Road, Xi'an, Shaanxi 710049, P. R. China.

Email: yxzhao@mail.xjtu.edu.cn

Experiment Section:

Apparatus

LAMP amplifications were run on SimpliAmp Thermo Cylers (Thermo Fisher Scientific Inc, USA). The real-time fluorescence measurements were performed with LightCycler 96 (Roche Applied Science, Mannheim, Germany). The electrophoresis were conducted on PowerPac Basic system (Bio-Rad, USA) and imaged by Syngene G:BOX Imaging System (Syngene System, Cambridge, UK). Centrifugation were carried out with ThermoSorvallST8R centrifuge (Thermo Fisher Scientific Inc, USA).

Materials and Reagents

All chemicals were obtained from commercial sources and used without further purification. Bst2.0 WarmStart ® DNA polymerase and 100mM magnesium sulfate solution were purchased from New England Biolabs Ltd. Betaine was obtained from Macklin Biochemical Company (Shanghai, China). dNTP and SYBR Green dye were ordered from Generay Biotechnology Co., Ltd (Shanghai, China). All oligonucleotides were ordered from Sangon Biotechnology Co., Ltd. (Shanghai, China). Oligonucleotide sequences are summarized in Table S1. All solutions were prepared and diluted with Milli-Q water (resistance 418.2 MΩ). The 380 bp conserved fragments of C gene in hepatitis B virus (HBV) were screened and cloned into a pUC57 vector by Sangon Biotechnology Co., Ltd. (Shanghai, China).

Sample preparation

1. Standard templates

pUC57 vector containing partial HBV C gene was commercially synthesize and 10-fold serially diluted to prepare standard template DNA with various concentrations (10^{-1} to 10^{8} copies/μL) in reaction system.

2. Clinical samples

With prior informed written consent, the human blood samples from 2 healthy volunteers and 8 HBV patients were obtained from the Second Affiliated Hospital of Xi'an Jiaotong University. The study was approved by the Institute Research Ethics Committee of The Second Affiliated Hospital.

1) Extract samples

Blood samples from healthy volunteers and HBV patients were firstly collected in coagulation-promoting tubes, centrifuged at 2000× g, and then supernatant serum was carefully collected. 300 μL serum was used for HBV genome extraction with a Viral genome DNA/RNA rapid extraction kit (Bioteke, Beijing, China), and a final elution volume of 30 μL was collected. Then 3 μL of elution volume was used for genome load qualification from this real sample.

2) Crude samples

Blood samples from healthy volunteers and HBV patients were firstly collected in coagulation-promoting tubes, centrifuged at 2000× g, and then supernatant serum was carefully collected. 1 μL serum was directly added in to LAMP reaction mixture without any pretreatments.

LAMP condition

Primers were designed using PrimerExplorer V5program at the website (http://primerexplorer.jp/e/) and used in the amounts typically recommended for LAMP: 0.2 μM each for outer primers F3 and B3; 1.2 μM each for inner primers FIP (F1-F2) and BIP (B1-B2); and 0.8 μM each for loop primers LF and LB.

LAMP reactions were performed in 30 μL reaction volumes in thin-walled PCR strip tubes. All primers with different copies of template were added into reaction mixture, which had a final composition of 1× ThermoPol reaction Buffer (20 mM Tris-HCl, 10 mM KCl, 10 mM $(NH_4)_2SO_4$, 2 mM $MgSO_4$ and 0.1% Triton X-100, pH 8.8) supplemented with an additional 4 mM $MgSO_4$ (final 6 mM $MgSO_4$), 0.4 mM each dNTP, 0.4 μM SYBR Green I and 1 M betaine. Mixtures were heated to 95 °C for 5 min to assist genome degeneration and primers annealing, followed by chilling on ice for 2 min. Then 4u Bst 2.0 WarmStart DNA polymerase was added to initiate the LAMP reaction. Reactions were incubated at a constant temperature of 65 °C for 60−120 min, with fluorescence read steps at intervals of 1 min. Incubation was typically followed by inactivation of the enzyme at 80 °C for 20 min.

Analysis of Reaction Products

1. Electrophoresis

Amplification products were analyzed by 3.5% agarose gel stained with GelRed DNA intercalating dye in 1×TBE buffer at 80 volt for 45 min.

2. Melting curve

Melting curve were obtained by continuous measurements fluorescence signal from 65 °C to 97 °C.

Table S1

Sequences of the DNA oligonucleotides involved in this study

Name		Sequences (5' to 3')	Usage
FIP		CTCCCGATACAGAGCAGAGGTTTGCCTTCTGACTTCTTTCC	Well-developed forward inner primer
BIP		TTGTTCACCTCACCATACAGCATGGGTCTTCCAAATTACTTCC	Well-developed backward inner primer
Op	F3	CTTCTGTGGAGTTACTCTCTT	Well-developed forward outer primer
	B3	GCTGACTACTAATTCCCTGG	Well-developed backward outer primer
Lp	LF	GGTGTCGAGGAGATCTCGAATA	Well-developed forward loop primer
	LB	TCTGTGTTGGGGTGAGTTGA	Well-developed backward loop primer
Op*	F3*	GACTTGGACTTGCTTCTGTGGAGTTACTCTCTT	CS labeled forward outer primer
	B3*	GACTTGGACTTGGCTGACTACTAATTCCCTGG	CS labeled backward outer primer
Lp*	12LF*	GACTTGGACTTGGGTGTCGAGGAGATCTCGAATA	12-mer CS labeled forward inner primer
	12LB*	GACTTGGACTTGTCTGTGTTGGGGTGAGTTGA	12-mer CS labeled backward inner primer
6LF*		GACTTGGGTGTCGAGGAGATCTCGAATA	6-mer CS labeled forward loop primer
6LB*		GACTTGTCTGTGTTGGGGTGAGTTGA	6-mer CS labeled backward loop primer
18LF*		GACTTGGACTTGGACTTGGGTGTCGAGGAGATCTCGAATA	18-mer CS labeled forward loop primer
18LB*		GACTTGGACTTGGACTTGTCTGTGTTGGGGTGAGTTGA	18-mer CS labeled backward loop primer
LF*		TCTGTGTTGGGGGGTGTCGAGGAGATCTCGAATA	Normalized forward loop primer
LB*		GGTGTCGAGGAGTCTGTGTTGGGGTGAGTTGA	Normalized backward loop primer
LF$^{\#}$		GACTTGGACTTGGGTGTCGAGGAGATCTCGAATA	Random sequences labeled forward loop primer

LB#	AGCTATAGCTATTCTGTGTTGGGGTGAGTTGA	Random sequences labeled backward loop primer
LF1*	ACAGAGCAGAGGGGTGTCGAGGAGATCTCGAATA	Janus forward loop primer normalized to inner sequence of FIP
LB1*	ACAGAGCAGAGGTCTGTGTTGGGGTGAGTTGA	Janus backward loop primer normalized to inner sequence of FIP
LF2*	CACCATACAGCAGGTGTCGAGGAGATCTCGAATA	Janus forward loop primer normalized to inner sequence of BIP
LB2*	CACCATACAGCATCTGTGTTGGGGTGAGTTGA	Janus backward loop primer normalized to inner sequence of BIP
LF3*	CTCCCGATACAGGGTGTCGAGGAGATCTCGAATA	Janus backward loop primer normalized to terminal sequence of FIP
LB3*	CTCCCGATACAGTCTGTGTTGGGGTGAGTTGA	Janus backward loop primer normalized to terminal sequence of FIP

Common sequences (CS) are underlined.

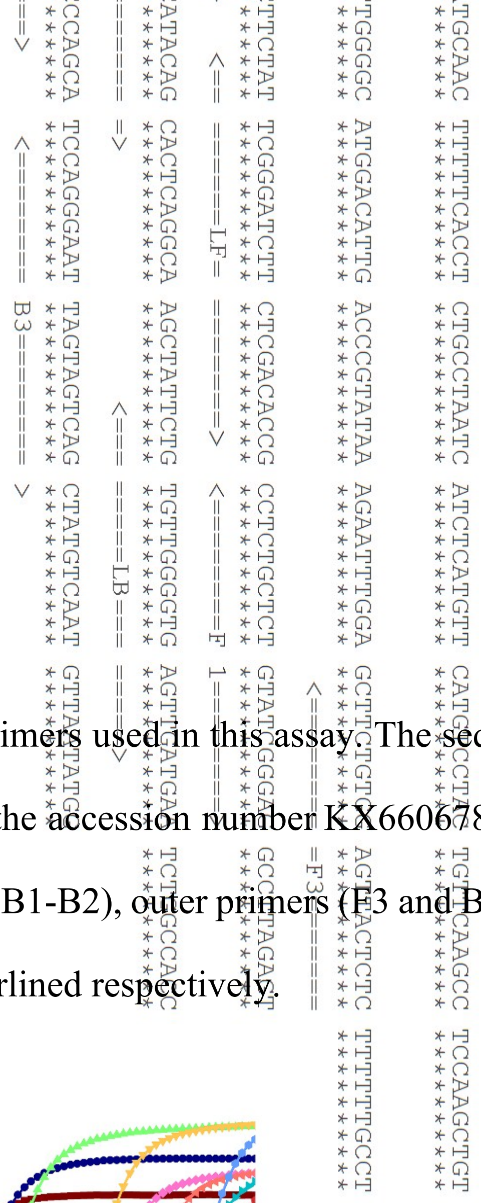

Fig. S1 The genomic position of primers used in this assay. The sequence was obtained from GenBank with the accession number KX660678. Two inner primers (FIP: F1-F2 and BIP: B1-B2), outer primers (F3 and B3) and loop primers (LF and LB) are underlined respectively.

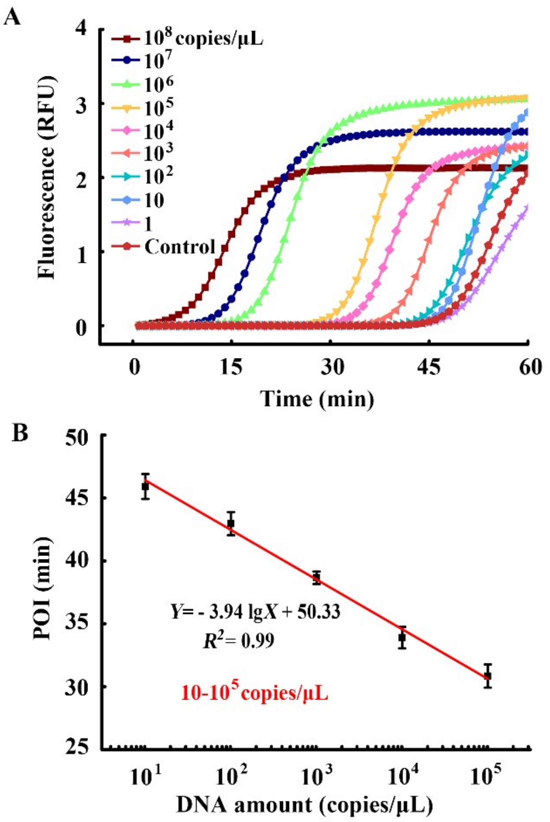

Fig. S2 (A) Real-time fluorescence signals of LAMP in well-developed system. From top to down, the amount of standard template DNA is 10^8,

10^7, 10^6, 10^5, 10^4, 10^3, 10^2, 10, 1 copies/μL and the blank control is conducted without template DNA. (B) The linear correlation between POI and the logarithm of template DNA amount ranging from 10 to 10^5 copies/μL.

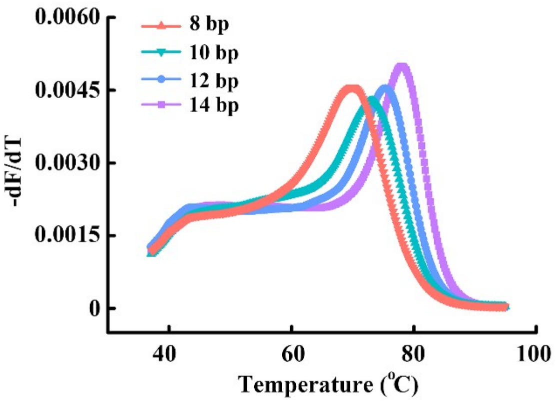

Fig. S3 The duplex stability of hairpin DNA with various stem length (8, 10, 12 and 14 bp) were investigated by melting curve analysis.

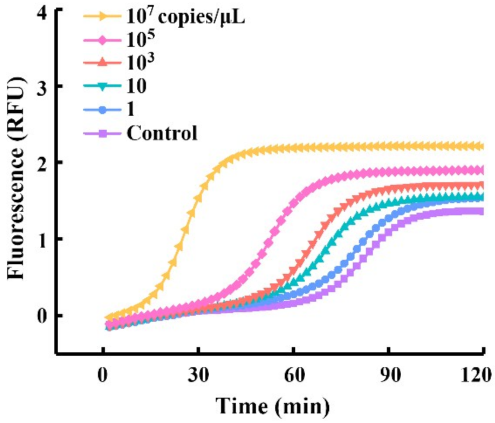

Fig. S4 Real-time fluorescence signals of LAMP reactions with normalized probes. From top to down, the amount of standard template DNA is 10^7, 10^5, 10^3, 10, 1 copies/μL and the blank control is conducted without template DNA.

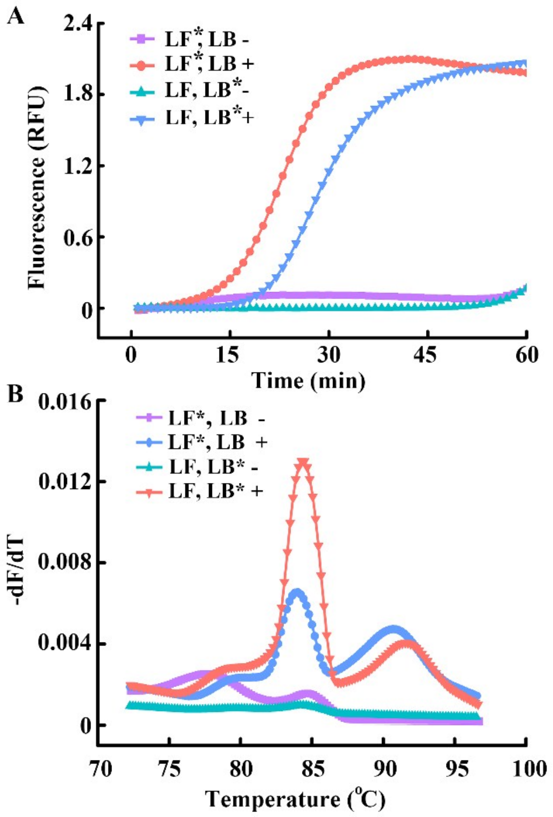

Fig. S5 (A) Real-time fluorescence signals of LAMP reactions with various normalized probes set. (B) Melting curve analysis of the amplifications with normalized probes. Asterisk (*) indicates the normalized probes. Plus and minus sign represent amplifications in the presence of 10^7 copies/μL template DNA and its corresponding backgrounds control without template, respectively.

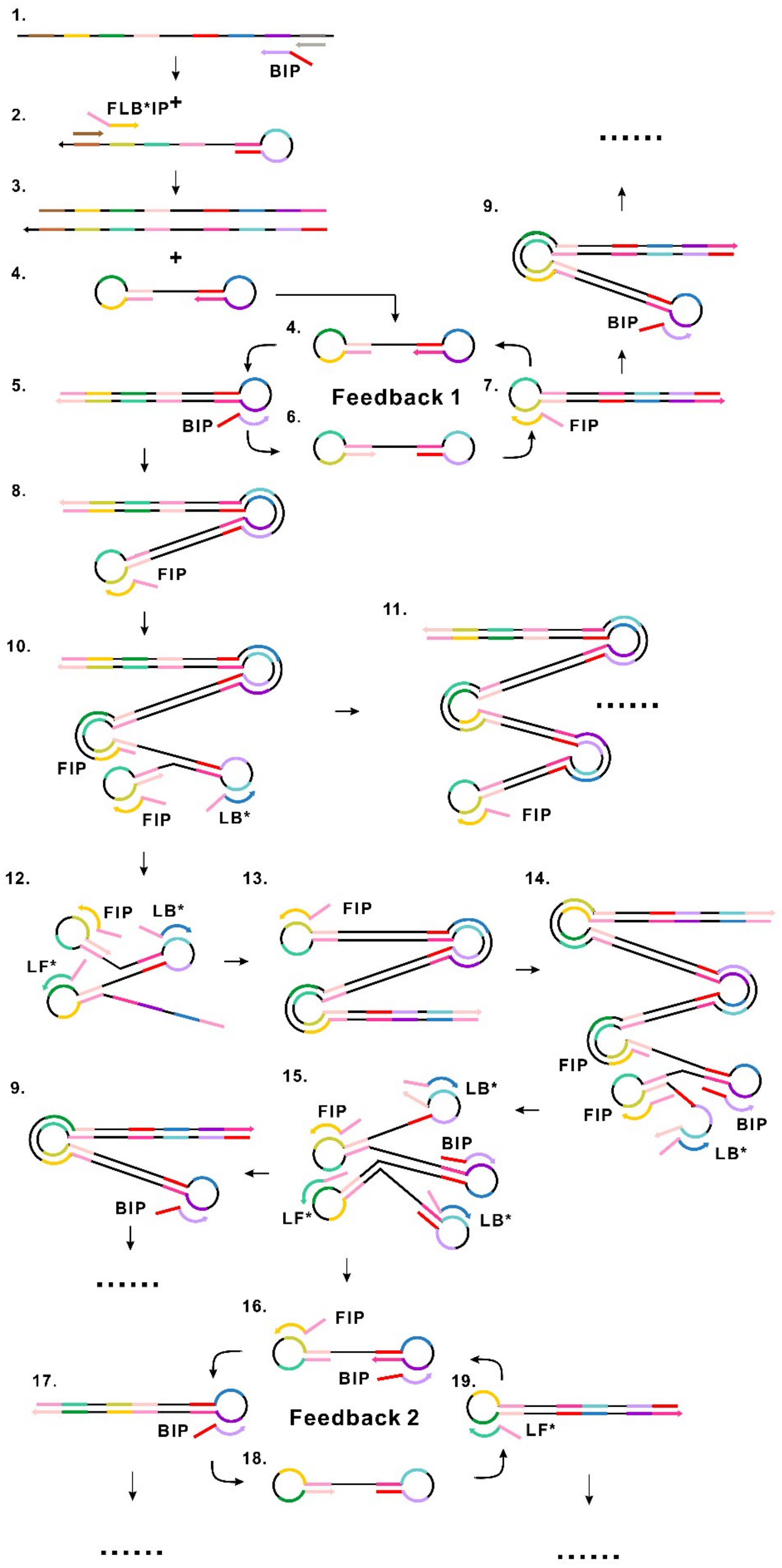

Fig. S6 Schematic illustration of engineered Janus probe mediated improved LAMP.

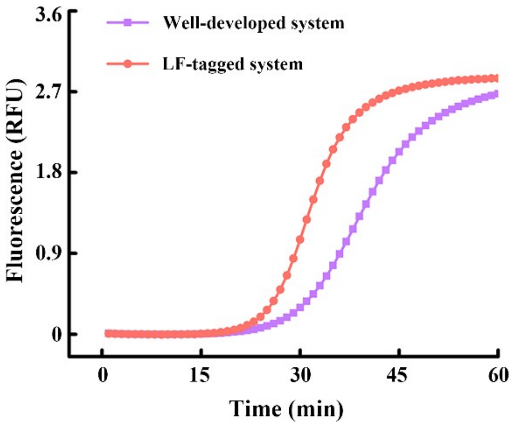

Fig.S7 Real-time fluorescence signals of LAMP reactions in different systems in presence of 10^5 copies/μL standard template DNA. In well-developed system, probe set contains six primers designed by program online. In LF-tagged system, LF primer tagged by the identical tails of FIP primer is used instead of original LF.

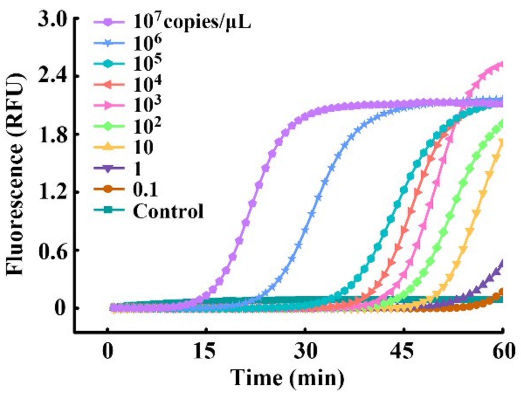

Fig. S8 Real-time fluorescence signals of LAMP reactions in Janus system. From top to down, the amount of standard template DNA is 10^7, 10^6, 10^5, 10^4, 10^3, 10^2, 10, 1, 0.1 copies/μL and the blank control is conducted without template DNA.

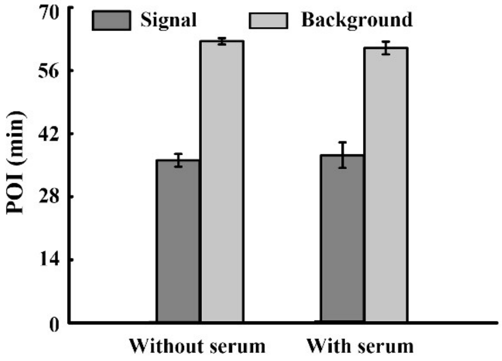

Fig. S9 Comparison of the amplification performance with and without introducing 1 microlitre serum. Signal is conducted in the presence 10^5 copies/μL template DNA with corresponding background controls without template.

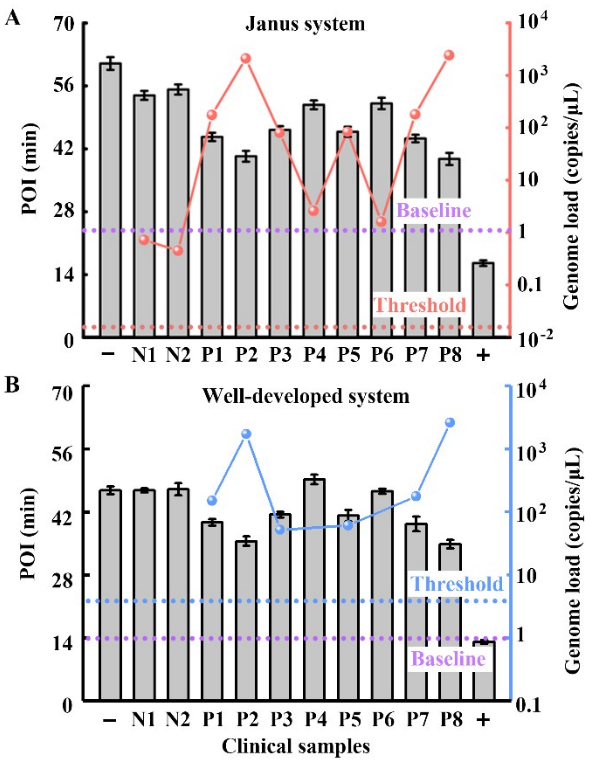

Fig. S10 Clinical assay performance of our Janus system (A) and well-developed system (B) for the quantification of HBV genome extracted from serums of patients and healthy volunteers using commercial kit. Plus and minus sign represent positive control in the presence of 10^7 copies/μL template DNA and its corresponding negative control without template. N1 and N2 represent samples from healthy volunteers, P1-8 represent samples from clinically confirmed positive patients. Threshold value (highlighted in red) is set at 0.03 (dash line in red) and 3 (dash line in blue) according to the dynamic range of each system and the addition of extracted samples. The baseline is defined as 1 copies/μL. Samples from to all patients can be distinguished clearly from healthy volunteers in Janus system, yet samples from patient 4 and 6 are undetectable by well-developed system.
